# Compound Probiotics Enhance Performance, Antioxidant Status, and Cecal SCFAs in Post-Molting Broiler Breeders

**DOI:** 10.3390/ani16010085

**Published:** 2025-12-28

**Authors:** Bowen Yang, Yuhan Wei, Yuqing Yang, Minhong Zhang, Chengmin Wang, Qi Wang, Jue Wang, Xuejing Wang, Baoliang Fan

**Affiliations:** 1 College of Animal Science and Technology, Hebei Agricultural University, Baoding 071000, China; yangbowen@hebau.edu.cn (B.Y.); weiyuhan502@163.com (Y.W.); 2 College of Animal Science and Technology, Hebei North University, Zhangjiakou 075132, China; y17325260729@outlook.com; 3 Hebei Institute of Animal Science and Veterinary Medicine, Baoding 071030, China; hbzhsyz123@163.com (Q.W.); wangjue55@163.com (J.W.); 4 Institute of Animal Science, Chinese Academy of Agricultural Sciences, Beijing 100193, China; zmh66@126.com; 5 Center for Wildlife Diseases and Immunology, Institute of Zoology, Guangdong Academy of Sciences, Guangzhou 510275, China; wangchm@giz.gd.cn

**Keywords:** compound probiotic, aging broiler breeders, post-molting period, antioxidant capacity, gut health

## Abstract

This study aimed to investigate the effects of a compound probiotic on production performance and antioxidant capacity in post-molting broiler breeders. A total of 6800 healthy 69-week-old Arbor Acres breeders were randomly allocated to 2 treatment groups (4 replicates of 850 birds each). Both groups were fed an identical basal diet, whereas the drinking water was supplemented with 75 g/t of the compound probiotic for the Experimental group only. The trial included a 7-day adaptation followed by a 49-day experimental period. Results demonstrated that drinking water supplementation with dietary compound probiotic preparation improved the laying performance rate, fertilization rate, and hatchability compared to the control (*p* < 0.05). The serum total antioxidant capacity was significantly enhanced (*p* < 0.05), while a concurrent trend of reduction in MDA content was also noted (*p* = 0.086). Significant decreases in serum total protein and globulin levels were observed (*p* < 0.05). Antioxidant profiles in reproductive tissues were also improved, with increased superoxide dismutase activity in the liver and oviduct, enhanced total antioxidant capacity in the ovary and oviduct, and a concurrent decrease in malondialdehyde content in the oviduct (*p* < 0.05). Furthermore, serum immunoglobulin M, and cecal butyric acid and isobutyric acid contents were higher in the compound probiotic preparation group (*p* < 0.05). In conclusion, supplementing drinking water with the compound probiotic during the post-molting period enhanced production performance, antioxidant status, and immune and gut health in aging broiler breeders, supporting the preservation of valuable poultry genetic resources.

## Simple Summary

Maintaining the health and productivity of aging broiler breeders is crucial for preserving valuable genetic lines in poultry farming. This study investigated whether adding a compound probiotic (75 g/t of drinking water) to drinking water could benefit breeders during the critical recovery phase after molting. The trial involved a large population of Arbor Acres breeders, with one group receiving the probiotic in their water. Results showed that the probiotic supplementation significantly enhanced laying performance, fertility, and hatchability. It also improved the birds’ overall antioxidant status, significantly elevated serum immunoglobulin M (IgM), and increased beneficial fatty acids in the cecum. These findings demonstrate that this probiotic strategy can effectively support the health and productivity of aging broiler breeders, offering a practical method to sustain elite poultry populations.

## 1. Introduction

Animal genetic resources are of strategic importance for ensuring national food security, promoting the sustainable development of the livestock industry, and safeguarding agricultural biodiversity [1]. As a pivotal genetic resource for global animal protein production, broiler breeders see their economic value and conversion efficiency directly influenced by the stability of their production performance, making their conservation and efficient utilization is a central challenge in modern animal husbandry [2]. In commercial production, however, egg production and egg quality decline as age [3], a process often exacerbated by the compromised antioxidant capacity of the birds due to accumulated reactive oxygen species (ROS) and free radicals [4]. Therefore, extending the productive lifespan of high-performing individuals represents a key strategy in animal production for maximizing their genetic potential and economic value, ultimately promoting the efficient utilization of elite genetic resources [5,6,7].

Induced molting is a pivotal management technique employed to rejuvenate aging broiler breeders, enabling a rapid recovery of physical condition and reproductive function to initiate a second highly productive laying cycle [8]. Nevertheless, this process itself induces significant physiological stress and can compromise intestinal health, creating a critical need for effective post-molting strategies. The post-molting period is thus a decisive stage for the determining the success of recovery in terms of subsequent egg production, fertilization rate, hatchability and other aspects [9]. Given the physiological challenges of this period, nutritional interventions that can mitigate stress and support recovery are of great interest.

Probiotics, recognized as safe and sustainable feed additives, have consequently garnered significant research interest and now widely applied in livestock and poultry production. Probiotics contribute to poultry health by modulating the gut microbiota, protecting gastrointestinal health, enhancing immunity, and consequently promoting growth performance [10]. Specific strains relevant to this study have demonstrated considerable benefits. For example, *Bacillus subtilis* can improve the production performance of broiler breeders, enhance egg quality and reproductive performance, strengthen immune function, and maintain the stability of intestinal microbiota [11]. Dietary supplementation with *Bacillus licheniformis* exerts beneficial effects on laying hens, including improved egg quality, enhanced intestinal health, and reinforced antioxidant and immune functions [12]. *Clostridium butyricum* plays a crucial role in broiler production. Its primary metabolite, butyric acid, helps maintain intestinal morphological integrity, improve growth performance, and enhance antioxidant capacity and immunity [13]. *Enterococcus faecalis* is beneficial for optimizing the cecal microbiota structure of broiler breeders and improving serum biochemical indices [14]. Compound probiotic preparations are processed from a combination of multiple probiotic strains, which can integrate the probiotic functions of each strain and synergistically promote animal growth and production performance [15]. Indeed, recent studies have indicated that compound probiotic can improve the production performance and immunity of broiler breeders [16]. However, while the general benefits of probiotics are established, their efficacy in mitigating the specific stresses of the post-molting period in aging broiler breeders, particularly concerning systemic and reproductive tract antioxidant capacity, remains less systematically investigated. Therefore, this study aimed to investigate the effects of a compound probiotic preparation—composed of *Bacillus subtilis*, *Bacillus licheniformis*, *Clostridium butyricum*, and *Enterococcus faecalis*—supplemented via drinking water on the production performance, antioxidant status, and key health parameters of broiler breeders during the post-molting period. The findings aim to provide a scientific basis for the application of this probiotic strategy, thereby evaluating its potential not only to enhance immediate production metrics but also to support the conservation of valuable genetic resources by extending the productive lifespan of high-performing flocks. The findings offer a practical strategy to enhance production performance and support genetic resource conservation by extending the productive lifespan of elite flocks.

## 2. Materials and Methods

### 2.1. Experimental Materials

The compound probiotic preparation consisted of four strains: *Bacillus subtilis* (6 × 10^9^ CFU/g), *Bacillus licheniformis* (4 × 10^9^ CFU/g), *Clostridium butyricum* (5 × 10^8^ CFU/g), and *Enterococcus faecalis* (1 × 10^9^ CFU/g). This probiotic product was provided by the Nanling Wanze Microbial Engineering Research Institute Co., Ltd., Nanling, China.

### 2.2. Experimental Design and Feeding Program

This experimental protocol was approved by the Institutional Animal Care and Use Committee of Hebei Agricultural University (Approval No. 2025116). All animal studies were conducted in accordance with the ARRIVE guidelines. A total of 6800 healthy 69-week-old Arbor Acres broilers with similar laying rates were randomly selected and divided into two groups: the Control group and the Experimental group. Each group included 4 replicates, with 850 hens per replicate. The compound probiotic preparation was administered via drinking water, with a supplementation level of 75 g/t [17] of drinking water in the Experimental group. The Control group was supplied with regular drinking water. The water quality provided throughout the experiment conformed to the Chinese agricultural standard for Hazard-Free Food–Water Quality for Livestock and Poultry Drinking (NY 5027-2008) [18]. The key parameters were as follows: pH 7.2 ± 0.3, total hardness 150 ± 20 mg/L (as CaCO_3_), residual chlorine < 0.05 mg/L, and total dissolved solids 280 ± 30 mg/L. All microbiological indicators met the sanitary requirements, thereby providing a stable aqueous medium for the probiotic formulation. Both groups were fed the same corn-soybean meal diet, formulated in accordance with the Feeding Standard of Chickens in China (NY/T 33-2004) [19]. The ingredient composition and nutrient levels of the experimental diet are presented in Table 1.

### 2.3. Rearing and Management

Induced molting was performed using a conventional feed withdrawal protocol. Briefly, hens were subjected to a 12-day period of complete feed withdrawal with free access to water. The lighting program was reduced to 8 h of light per day during this period. The flock achieved a target body weight loss of 25–30%, with process mortality maintained below 5%. Molting was considered successfully initiated when target weight loss was reached and the shedding of primary wing feathers was observed. The trial probiotic supplementation began immediately thereafter in the recovery period. After the induced molt, followed by the recovery of egg production rate to >20%, the birds entered a 7-day adaptation period prior to the 49-day formal experiment. During the experiment, broiler breeders were housed in a litter-based floor-rearing system with consistent environmental conditions. The stocking density was 15 birds/m^2^. Feed restriction was implemented, with an average feed intake of 150 g per hen daily. Roosters accounted for 7% of the total flock for natural mating. The poultry houses adopted negative-pressure ventilation, and the temperature was maintained at approximately 26 °C. Hens were fed twice daily at 8:00 and 15:00, with ad libitum access to clean drinking water. Artificial lighting was provided for a total of 16 h daily. Vaccinations were administered following standard routine procedures. To maximize the viability of probiotics in the drinking water system, the following management measures were implemented. The probiotic-supplemented water was prepared fresh immediately before the morning and evening feeding times. A precise amount of probiotic powder was completely dissolved in clean lukewarm water to prepare a stock solution, which was then thoroughly mixed into the drinking water system. The water lines were flushed prior to each addition. The prepared probiotic water was ensured to be consumed by the birds within 4 h to maximize the viability of the probiotic strains.

### 2.4. Assays and Analytical Methods

#### 2.4.1. Laying Performance

After the experiment started, the number of eggs laid, egg weight, and number of substandard eggs were recorded daily for each replicate. Feed consumption was calculated weekly by accounting for residual feed. At the end of the experiment, the laying performance rate, average egg weight, and feed conversion ratio (FCR) were calculated for each group. The calculation methods for laying performance rate, average egg weight, egg mass and FCR are as follows:

Laying performance rate (%)=(total number of eggs)/(number of laying hens×number of days)×100%

Average egg weight (g)=total egg mass/total number of eggs

Egg mass (g/d)=laying performance rate×average egg weight

FCR=total feed intake/total egg mass

#### 2.4.2. Sample Collection

To ensure the samples were representative of each replicate, a balanced sampling strategy was employed across all four replicates within a treatment group. At the end of the experiment, a total of 32 eggs per treatment group were randomly selected and stored at 4 °C for egg quality analysis. These eggs were evenly sampled from the four replicates, resulting in 8 eggs collected from each replicate. Similarly, a total of 60 eggs per treatment group were randomly selected for the assessment of reproductive performance. This was achieved by evenly collecting 15 eggs from each of the four replicates. Prior to sampling on the final day, hens were fasted for 12 h. A total of eight hens per treatment group (2 hens randomly selected from each replicate) were used for the collection of blood, tissue, and cecal content samples. Blood samples were collected from the wing vein using sterile venipuncture techniques. The blood was placed in serum separator tubes, allowed to clot at room temperature for 30 min, and then centrifuged at 1500× *g* for 10 min to separate the serum. The supernatant serum was aliquoted into sterile centrifuge tubes and immediately stored at −20 °C for subsequent analysis. After blood collection, the same eight hens were humanely euthanized by cervical dislocation. Tissue samples, including the liver, ileum mucosa, ovary, and oviduct, were promptly collected. Immediately following tissue collection, cecal contents were aseptically gathered from the same hens. All tissue and cecal content samples were flash-frozen in liquid nitrogen and subsequently transferred to a −80 °C freezer for long-term storage until analysis.

#### 2.4.3. Egg Quality

The length and width were measured using a vernier caliper to calculate the egg shape index. Eggshell strength was determined with the Egg Force Reader (Orka Food Technology Co., Ltd., Ramat HaSharon, Israel). Yolk weight was measured using an electronic balance to calculate the yolk ratio. Shell thickness was measured with a micrometer. Albumen height, Haugh unit and yolk color were assessed by the Egg Analyzer (Orka Food Technology Co., Ltd., Ramat HaSharon, Israel).

#### 2.4.4. Reproductive Performance

At the end of the experiment, 60 eggs were incubated in a standard incubator to determine the reproductive performance. The fertility rate and healthy chick rate were calculated based on the counts of hatching eggs, fertile eggs, unfertilized eggs, and hatched chicks on the 21st day of incubation. All chicks were weighed and then euthanized. The total weights of the stomach (including both the glandular stomach and the muscular stomach) and intestines were measured, followed by the calculation of the stomach index and intestinal index. The stomach and intestinal indices were calculated as a percentage of chick hatch weight: (Organ Weight/Hatch Weight) × 100.

#### 2.4.5. Serum Index

The contents of total protein (TP), albumin (ALB), globulin (GLB), total cholesterol (TC), and triglycerides (TG) in serum were determined using assay kits. The activities of superoxide dismutase (SOD) and glutathione peroxidase (GSH-Px), as well as the content of malondialdehyde (MDA) and total antioxidant capacity (T-AOC) in serum were determined using assay kits. All kits were purchased from Nanjing Jiancheng Bioengineering Institute, Najing, China.

#### 2.4.6. Tissue Antioxidant Capacity

All samples were homogenized, and the supernatant was collected for analysis. To account for tissue variation, indicators in tissue samples were normalized to the total protein content. This normalization is standard practice as it corrects for variations in tissue cellularity and homogenate concentration, thereby expressing the activity or concentration of the analyte per unit of cellular material. The total protein content was determined by the bicinchoninic acid (BCA) method. The activities of SOD and GSH-Px, the content of MDA, and T-AOC were determined using assay kits (Nanjing Jiancheng Bioengineering Institute, Nanjing, China).

#### 2.4.7. Serum and Ileal Immune Indicators

The levels of immunoglobulins (IgA, IgG, IgM) in serum and IgA and secretory immunoglobulin A (SIgA) in ileal mucosa were determined using ELISA kits. Ileal mucosal samples were homogenized, and the supernatant was collected for analysis. To account for tissue variation, immunoglobulin concentrations in mucosal samples were normalized to the total protein content, which was determined by the BCA method. All kits were purchased from Nanjing Jiancheng Bioengineering Institute (Nanjing, China).

#### 2.4.8. Short-Chain Fatty Acid Content in Cecum

The types and concentrations of short-chain fatty acids (SCFAs) in the cecal content were quantified using gas chromatography–mass spectrometry (GC-MS) according to the method described by Malhi et al. [20] Briefly, approximately 1.5 g of the sample was accurately weighed and homogenized with normal saline at a 1:2 (*m*/*v*) ratio. The mixture was vortexed thoroughly and centrifuged at 2000× *g* for 15 min. Subsequently, 1 mL of the supernatant was transferred to a 1.5 mL microcentrifuge tube, kept at 4 °C for 30 min, and then centrifuged at 2500× *g* for 20 min. The resulting supernatant was injected into the GC-MS system (Agilent Technologies Inc., Santa Clara, CA, USA) for analysis. Separation was performed on a DB-FFAP capillary column (60 m × 250 μm × 0.5 μm, Agilent Technologies Inc., Santa Clara, CA, USA). Qualitative analysis of fatty acids was conducted using the Qualitative Analysis software (ver. b.07.00) by comparing mass spectra against the NIST 14.0 mass spectral library. Quantification of SCFAs was achieved using the Agilent Mass Hunter 11.0 software based on external standard calibration curves prepared from serially diluted standards. The results were expressed as μg per gram of wet cecal content (μg/g).

### 2.5. Data Analysis

All data were analyzed using independent-samples *t*-test with SPSS 26.0 software. Results were expressed as mean ± standard deviation (SD). A value of *p* < 0.05 was considered statistically significant. Data for laying performance rate were analyzed using a repeated-measures analysis of variance (ANOVA) to appropriately account for the longitudinal nature of the measurements collected from the same experimental units across multiple weeks. The model included Treatment (Control vs. Probiotic) as a between-subjects factor and Time (Week) as a within-subjects factor. The assumption of sphericity was tested using Mauchly’s test. Since the assumption was violated (*p* < 0.001), the degrees of freedom for within-subjects effects were corrected using the Greenhouse–Geisser estimate of sphericity (ε = 0.259). The main effects of Treatment and Time, as well as their interaction (Treatment × Time), were examined. For between-subjects effects (Treatment), significance was assessed at *p* < 0.05.

## 3. Results and Analysis

### 3.1. Laying Performance

According to Table 2, supplementation of the compound probiotic preparation in drinking water significantly increased the laying performance rate of broiler breeders during the post-molting period (*p* < 0.05). Average egg weight, egg mass, and feed conversion ratio showed no significant changes (*p* > 0.05).

**Figure 1:**
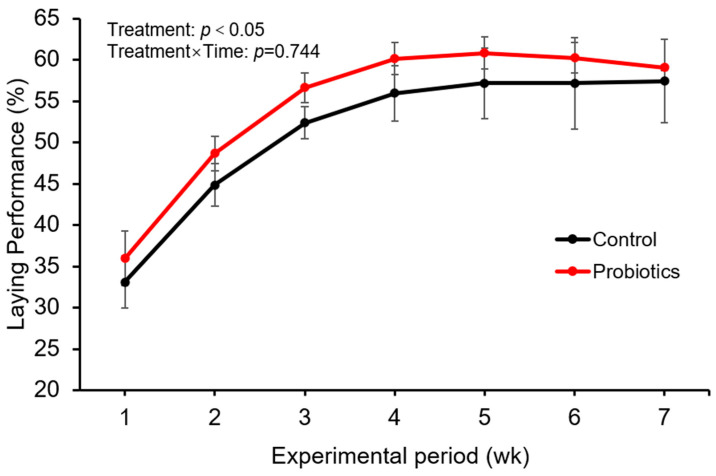
Change in Egg Production Rate During the Experimental Period (*n* = 4). Data are presented as mean ± standard deviation. Data were analyzed by repeated-measures ANOVA. The main effect of treatment (Probiotic vs. Control) was significant (F(1,6) = 6.826, *p* = 0.040, where the values in parentheses denote degrees of freedom for the effect and error, respectively). The interaction between treatment and time was not significant (*p* = 0.744), indicating a consistent treatment effect across weeks.

The effects of probiotic supplementation on post-molting laying performance are presented in Figure 1. A repeated-measures ANOVA revealed a significant main effect of Time (*p* < 0.001), reflecting the expected recovery of egg production across weeks following induced molting. More importantly, a significant main effect of Treatment was observed (F(1,6) = 6.826, *p* = 0.040), with the probiotic group maintaining a higher overall laying rate compared to the control. The Treatment × Time interaction was not significant (*p* = 0.744), indicating that the beneficial effect of the probiotic remained consistent throughout the experimental period.

### 3.2. Egg Quality

As shown in Table 3, supplementation of the compound probiotic preparation in drinking water had no significant effect on the egg quality of broiler breeders during the post-molting period (*p* > 0.05).

### 3.3. Reproductive Performance

Dietary supplementation with the compound probiotic contributed to enhanced reproductive performance in broiler breeders, with significant improvements in both fertility rate and total hatchability (*p* < 0.05, Table 4). Furthermore, this intervention led to a significant improvement in the intestinal development of the offspring, as indicated by a higher intestinal index (*p* < 0.05). All other measured parameters for both breeders and chicks remained comparable between the groups.

### 3.4. Serum Biochemical Indices

The supplementation of the compound probiotic via drinking water significantly modulated specific serum biochemical parameters (Table 5). Notably, it led to a reduction in TP and GLB levels (*p* < 0.05). In contrast, ALB, TG, and TC levels were not significantly influenced by the treatment (*p* > 0.05).

### 3.5. Serum Antioxidant Capacity

The compound probiotic supplementation markedly improved the systemic antioxidant status of broiler breeders during post-molting, as evidenced by a significant increase in serum T-AOC (*p* < 0.05). In addition, a decreasing trend in MDA content was observed, further indicating a beneficial effect (Table 6). This enhancement occurred independently of changes in the key antioxidant enzymes SOD and GSH-Px, whose activities remained unaltered.

### 3.6. Tissue Antioxidant Capacity

As shown in Table 7, the supplementation of the compound probiotic in drinking water differentially modulated the antioxidant capacity across various tissues. In the liver, it significantly increased the activity of SOD (*p* < 0.05) without affecting other measured indices. In the ovary, a significant enhancement in the T-AOC was observed (*p* < 0.05), with no other significant changes. The most pronounced effects were found in the oviduct, where the probiotic significantly increased T-SOD activity and T-AOC while concurrently reducing the MDA content (*p* < 0.05). In contrast, none of the antioxidant indices in the ileum were significantly altered by the treatment.

### 3.7. Serum and Ileal Mucosa Immune Indices

The immunomodulatory effect of the compound probiotic was specific to systemic IgM production, as shown in Table 8. Supplementation significantly elevated serum IgM levels (*p* < 0.05), indicating a targeted enhancement in this arm of the humoral immune response. However, this effect did not extend to other serum immunoglobulins (IgA, IgG) or to the local mucosal immune response in the ileum, where IgA and SIgA contents remained unchanged.

### 3.8. Cecal Short-Chain Fatty Acids Contents

Supplementation with the compound probiotic selectively modulated the cecal SCFAs profile by significantly enhancing the production of butyric acid and isobutyric acid (*p* < 0.05, Table 9). Furthermore, strong tendencies toward increases were observed for acetic acid (*p* = 0.088) and 4-methylvaleric acid (*p* = 0.083). The concentrations of other SCFAs were not significantly altered by the probiotic supplementation.

## 4. Discussion

Currently, substantial research has been conducted on the effects of probiotics on animal production performance. The compound probiotic preparation utilized in this study consisted of *Bacillus subtilis*, *Bacillus licheniformis*, *Clostridium butyricum*, and *Enterococcus faecalis*, which function synergistically. In contrast to previous studies that predominantly focused on single-strain probiotics or dietary supplementation methods [21,22], the present study innovatively evaluated a multi-strain probiotic complex administered via drinking water. During the critical stress period of post-molting, this intervention demonstrated considerable potential for enhancing the overall performance of broiler breeders, providing a practical and effective nutritional strategy. The results indicated that supplementing drinking water with the compound probiotic preparation significantly improved the laying rate of broiler breeders during the post-molting period, thereby enhancing production performance. These findings are consistent with earlier reports by Salem et al. [23] and Zhao et al. [24] The underlying mechanisms may involve, but are not limited to, enhanced nutrient digestion and improved gut health. For instance, *Bacillus subtilis* and *Bacillus licheniformis* can produce a variety of digestive enzymes that promote nutrient digestion and absorption [25,26]. Meanwhile, the inclusion of *Clostridium butyricum* and *Enterococcus faecalis* synergistically enhanced this effect by complementing the spectrum of digestive enzymes [27]. Additionally, the compound probiotic preparation may improve intestinal development and maintain gut health through multiple pathways, ultimately enhancing nutrient utilization and production performance.

Optimal reproductive performance during the post-molting period is crucial for economic returns in broiler breeders. This study revealed that supplementation with the compound probiotic preparation significantly increased the fertilization rate and hatching rate of broiler breeders, and significantly improved the intestinal index of offspring chicks. These results are consistent with those reported by Lebedev et al. [28] and Qu et al. [29] The improvement in fertilization rate may be attributed to probiotic-induced regulation of reproductive hormone secretion and an enhanced follicular development microenvironment [30]. The significant improvement in hatchability observed in this study largely reflects the direct result of the enhanced fertility rate. This is a key finding as it directly correlates with economic returns. However, it is also acknowledged that hatchability is a composite metric, jointly influenced by both fertilization success and the survival rate of embryos in fertilized eggs. Therefore, hatchability holds indispensable practical value as a key performance indicator for assessing overall production efficiency. Furthermore, the probiotic may have improved male fertility. The anti-inflammatory and antioxidant effects conferred to the hens could have been transferred to the roosters via direct contact during natural mating or via the environment, potentially improving semen quality, sperm motility, and viability by reducing oxidative damage to sperm cells. The enhancement likely involved improved female reproductive efficiency. The significantly improved antioxidant capacity in the oviduct (increased SOD and T-AOC, decreased MDA) would create a more favorable microenvironment for sperm storage, survival, and function within the hen’s sperm storage tubules (SSTs). This could prolong sperm viability and increase the likelihood of successful fertilization. Therefore, the probiotic’s effect on fertility is likely mediated through a combination of improved sperm quality and an optimized oviductal environment for fertilization.

A notable finding of this study was the significant reduction in serum TP and GLB levels in the probiotic-supplemented group. While serum albumin concentration remained unchanged, the decrease in GLB, may indicate an alleviation of systemic inflammatory status [31]. This potential mitigation of inflammation could be attributed to the anti-inflammatory properties of probiotic-derived metabolites, such as butyrate [32].

Age-related structural changes in hepatocytes can exacerbate oxidative stress. In aging laying hens, the level of ROS increases with age, while the capacity of the endogenous antioxidant system to neutralize free radicals and peroxides declines [33]. This study revealed that supplementing drinking water with the compound probiotic preparation significantly improved systemic and tissue antioxidant status, as evidenced by increased T-AOC and T-SOD activities in serum, liver, ovary, and oviduct, with a concomitant trend toward reduction in MDA levels, and elevated serum IgM levels in post-molting broiler breeders. These observations are consistent with the findings of Bai et al. [34], Zhao et al. [35], and Zhang et al. [36] The collective improvement in antioxidant parameters across serum, liver, and reproductive organs indicates that probiotic supplementation effectively alleviated oxidative stress during the post-molting period. T-AOC serves as a comprehensive biomarker reflecting the non-specific and integrated antioxidant defense status in animals [37]. The observed enhancement in T-AOC indicates that the probiotic supplementation conferred a systemic, broad-spectrum antioxidant benefit. This effect may be mediated through indirect mechanisms involving the modulation of gut microbiota homeostasis by the compound probiotic preparation, leading to reduced production of endogenous oxidants. Concurrently, specific probiotic strains contributed via distinct pathways: *Bacillus subtilis* directly eliminated free radicals through the production of antioxidant enzymes such as superoxide dismutase and catalase [38], while *Bacillus licheniformis* upregulated the Nrf2 signaling pathway to enhance the expression of antioxidant enzymes [39,40]. Moreover, butyrate, produced by *Clostridium butyricum*, likely played a pivotal role by promoting the transcription of antioxidant-related genes via epigenetic regulation and activating the Nrf2/ARE signaling pathway, thereby upregulating downstream antioxidant enzymes [41,42,43]. The significant increase in serum IgM levels suggests that the probiotics may enhance antioxidant status indirectly through immunomodulation. It is well established that appropriate immune activation can strengthen the body’s antioxidant defense capacity, whereas excessive immune responses may aggravate oxidative stress [44]. The moderate elevation in IgM observed in this study likely reflects a beneficial immunomodulatory effect induced by the probiotic supplementation. Although the present study confirmed the antioxidant benefits of the compound probiotic, the precise underlying pathways remain to be fully elucidated. Future studies should employ integrated transcriptomic and metabolomic approaches (e.g., RNA-seq and LC-MS/GC-MS, respectively) to clarify the molecular mechanisms by which these probiotics regulate the antioxidant system, with particular emphasis on the synergistic interactions among the constituent bacterial strains.

The cecum serves as the primary site for microbial fermentation in poultry and plays a crucial role in SCFA production [45]. SCFAs function as key mediators of host-microbiota crosstalk, providing energy to the host and promoting overall health [46]. This study found that supplementation with the compound probiotic preparation during the post-molting period significantly increased the concentrations of SCFAs such as butyrate and isobutyrate in the cecum of broiler breeders. This result is in agreement with reports by Xu et al. [47] and Śliżewska et al. [48] Butyrate, an essential microbial metabolite [49], is critical for maintaining intestinal barrier integrity and functions as an important signaling molecule with anti-inflammatory and epigenetic regulatory properties. The notable increase in cecal butyrate levels is likely due to probiotic-induced enhancement in the abundance and metabolic activity of butyrate-producing bacteria within the cecal microbiota [50]. Previous studies have demonstrated that *Clostridium butyricum* directly synthesizes butyrate and promotes the proliferation of other butyrate-producing bacteria via cross-feeding mechanisms [51,52]. *Enterococcus faecalis* produces lactate, a metabolic precursor that fuels the growth and butyrate-synthesizing activity of butyrate-producing bacteria through cross-feeding [53,54]. Furthermore, *Bacillus subtilis* and *Bacillus licheniformis* improve the intestinal environment, thereby facilitating the colonization and metabolic function of *Clostridium butyricum* [55,56]. Therefore, the efficacy of the composite probiotic arises not only from the additive effects of individual strains but also from bacterial interactions that collectively activate the endogenous butyrate-producing metabolic network. Consistent with Zeng et al. [57] but contrasting with Zhang et al. [58], probiotic supplementation did not significantly alter the concentrations of other cecal SCFAs, such as acetate and propionate. These discrepancies may be attributed to differences in animal physiological stage, probiotic strain specificity, and dosage. This suggests that the compound probiotics modulate microbial metabolism in a highly specific manner, and the selective stimulation of butyrate production underlies their unique efficacy.

However, a potential limitation of this study is the lack of quantitative assessment of the dynamic survival rate of probiotics within the drinking system. The significant physiological improvements observed provide strong indirect evidence that viable probiotics were successfully delivered and functionally active. Furthermore, the probiotic product used in this study exhibited high stability (100% solubility and stable viable counts ≥ 10^10^ CFU/g), and the inclusion of *Bacillusstrains* in spore form ensured strong environmental tolerance. Combined with strict water quality control and fresh preparation practices, these factors collectively supported the effective delivery of probiotics. Future studies should incorporate regular monitoring of viable bacterial counts at the endpoint of drinking lines to more precisely quantify the actual dosage ingested by the birds.

## 5. Conclusions

Our study demonstrates that supplementing the compound probiotic in drinking water post-molting effectively enhances the productivity and health of aging broiler breeders. The intervention boosted antioxidant status, immune parameters, and cecal SCFAs profiles, leading to improved laying and reproductive performance. By mitigating age-related declines, this probiotic strategy supports the extended utilization of elite genetic lines, contributing directly to the conservation of valuable poultry genetic resources.

## Figures and Tables

**Table 1:** Ingredient Composition and Nutrient Levels of the Experimental Diet (%).

Ingredients	Content (%)	Nutrients ^2^	Content (%)
Corn	65.20	Metabolizable Energy (ME)	11.93
Soybean Meal	21.20	Crude protein (CP)	16.50
Wheat	3.00	Calcium	3.00
Limestone	6.92	Total phosphorus	0.70
Dicalcium phosphate	1.82	Non-phytate phosphorus	0.40
Sodium Bicarbonate	0.30	Digestible Lysine	0.68
Salt	0.26	Digestible Methionine	0.39
Premix ^1^	1.00	Digestible Threonine	0.55
DL-Methionine	0.26		
L-Lysine Hydrochloride	0.04		
Total	100.00		

^1^ The premix provided the following per kg of diet: Vitamin A 200,000 IU; Vitamin D_3_ 275,000 IU; Vitamin E (VE) 1490 IU; Vitamin K_3_ (VK_3_) 100 mg; Vitamin B_2_ (VB_2_) 1250 mg; Vitamin B_6_ (VB_6_) 60 mg; Vitamin B_12_ (VB_12_) 0.5 mg; niacin 600 mg; folic acid 8 mg; calcium pantothenate 600 mg; iron (Fe) 8000 mg; copper (Cu) 800 mg; manganese (Mn) 6000 mg; selenium (Se) 35 mg; iodine (I) 60 mg; choline chloride 50 g; DL-methionine 129 g. ^2^ Metabolizable energy (ME) was a calculated value. Others were measured values.

**Table 2:** Effects of Compound Probiotic Preparation on Laying Performance of Broiler Breeders During Post-Molting Period ^1^.

Items	Control Group	Probiotic Group	*p*-Value
Laying Performance Rate (%)	51.14 ± 2.06 ^b^	55.00 ± 1.55 ^a^	0.040
Average Egg Weight (g)	69.40 ± 0.94	67.48 ± 2.42	0.188
Egg Mass (g/d)	35.48 ± 1.27	36.80 ± 2.27	0.351
Feed Conversion Ratio	4.23 ± 0.15	4.09 ± 0.25	0.367

^1^ Data represent the mean value of 4 replicates each treatment (*n* = 4). ^a,b^ Means within a row with no common superscripts differ significantly (*p* < 0.05).

**Table 3:** Effects of Compound Probiotic Preparation on Egg Quality of Broiler Breeders During Post-Molting Period ^1^.

Items	Control Group	Probiotic Group	*p*-Value
Egg Shape Index	1.32 ± 0.09	1.31 ± 0.10	0.935
Eggshell Strength (N)	32.24 ± 8.70	34.98 ± 9.43	0.346
Albumen Height (mm)	6.94 ± 1.04	6.66 ± 0.95	0.388
Haugh Unit	79.55 ± 8.78	78.75 ± 6.91	0.751
Yolk Color	11.95 ± 2.00	11.70 ± 0.89	0.606
Yolk Ratio (%)	31.84 ± 1.57	31.75 ± 2.13	0.889
Eggshell Thickness (mm)	0.32 ± 0.03	0.31 ± 0.03	0.731

^1^ Data represent the mean value of 30 eggs each treatment (*n* = 32).

**Table 4:** Effects of Compound Probiotic Preparation on Reproductive Performance of Broiler Breeders and Chick Development During Post-Molting Period ^1^.

Items	Control Group	Probiotic Group	*p*-Value
Fertility Rate (%)	75.00 ± 1.92 ^b^	83.33 ± 2.72 ^a^	0.002
Hatchability (%)	58.33 ± 5.77 ^b^	71.67 ± 5.77 ^a^	0.017
Hatchability of Fertile Eggs (%)	77.96 ± 9.70	86.19 ± 9.15	0.263
Healthy Chick Rate (%)	94.24 ± 0.57	96.41 ± 2.41	0.130
Hatch Weight of Chicks (g)	48.70 ± 4.44	48.35 ± 3.18	0.776
Stomach Index of Chicks	4.92 ± 0.56	5.16 ± 0.94	0.333
Intestinal Index of Chicks	3.38 ± 0.67 ^b^	3.95 ± 0.80 ^a^	0.019

^1^ Data represent the mean value of 60 eggs each treatment (*n* = 60). No data points were removed as outliers during statistical analysis. The standard deviation reflects the biological variability within the replicates. ^a,b^ Means within a row with no common superscripts differ significantly (*p* < 0.05).

**Table 5:** Effects of Compound Probiotic Preparation on Serum Biochemical Indices of Broiler Breeders During Post-Molting Period ^1^.

Items	Control Group	Probiotic Group	*p*-Value
TP (g/L)	69.21 ± 7.61 ^a^	55.87 ± 6.18 ^b^	0.002
ALB (g/L)	19.57 ± 2.04	19.67 ± 1.52	0.904
GLB (g/L)	49.64 ± 8.89 ^a^	36.20 ± 5.00 ^b^	0.002
TG (mmol/L)	4.69 ± 4.23	4.79 ± 3.79	0.959
TC (mmol/L)	3.76 ± 1.34	3.33 ± 0.71	0.442

TP = total protein; ALB = albumin; GLB = globulin; TC = total cholesterol; TG = triglycerides. ^1^ Data represent the mean value of 8 hens each treatment (*n* = 8). ^a,b^ Means within a row with no common superscripts differ significantly (*p* < 0.05).

**Table 6:** Effects of Compound Probiotic Preparation on Serum Antioxidant Capacity of Broiler Breeders During Post-Molting Period ^1^.

Items	Control Group	Probiotic Group	*p*-Value
SOD (U/mL)	245.66 ± 75.01	228.70 ± 67.70	0.642
GSH-Px (U/mL)	573.52 ± 129.59	542.22 ± 90.13	0.584
MDA (μmol/L)	10.05 ± 1.59	8.67 ± 1.19	0.086
T-AOC (U/mL)	0.62 ± 0.08 ^b^	1.10 ± 0.29 ^a^	0.001

SOD = superoxide; GSH-Px = glutathione peroxidase; MDA = malondialdehyde; T-AOC = total antioxidant capacity. ^1^ Data represent the mean value of 8 hens each treatment (*n* = 8). ^a,b^ Means within a row with no common superscripts differ significantly (*p* < 0.05).

**Table 7:** Effects of Compound Probiotic Preparation on Tissue Antioxidant Capacity of Broiler Breeders During Post-Molting Period ^1^.

Items	Control Group	Probiotic Group	*p*-Value
Liver			
SOD (U/mg prot)	376.25 ± 92.22 ^b^	548.94 ± 76.98 ^a^	0.001
GSH-Px (U/mg prot)	18.12 ± 3.05	19.92 ± 6.18	0.472
MDA (μmol/g prot)	0.56 ± 0.32	0.56 ± 0.34	0.964
T-AOC (U/mg prot)	0.16 ± 0.03	0.16 ± 0.03	0.572
Ovary			
SOD (U/mg prot)	168.48 ± 66.51	176.22 ± 42.45	0.785
GSH-Px (U/mg prot)	41.97 ± 12.09	45.34 ± 9.68	0.548
MDA (μmol/g prot)	1.24 ± 1.21	1.00 ± 0.40	0.614
T-AOC (U/mg prot)	0.13 ± 0.06 ^b^	0.19 ± 0.05 ^a^	0.019
Oviduct			
SOD (U/mg prot)	176.63 ± 28.72 ^b^	263.22 ± 61.96 ^a^	0.003
GSH-Px (U/mg prot)	114.39 ± 28.33	112.99 ± 19.42	0.356
MDA (μmol/g prot)	0.89 ± 0.43 ^a^	0.49 ± 0.21 ^b^	0.035
T-AOC (U/mg prot)	0.14 ± 0.05 ^b^	0.21 ± 0.05 ^a^	0.023
Ileum Mucosa			
SOD (U/mg prot)	276.70 ± 62.42	246.39 ± 105.40	0.495
GSH-Px (U/mg prot)	32.74 ± 12.97	22.22 ± 10.36	0.095
MDA (μmol/g prot)	0.44 ± 0.19	0.70 ± 0.33	0.076
T-AOC (U/mg prot)	0.15 ± 0.03	0.17 ± 0.07	0.451

^1^ Data represent the mean value of 8 hens each treatment (*n* = 8). ^a,b^ Means within a row with no common superscripts differ significantly (*p* < 0.05).

**Table 8:** Effects of Compound Probiotic Preparation on Serum and Ileal Mucosa Immune Indices of Broiler Breeders During Post-Molting Period ^1^.

Items	Control Group	Probiotic Group	*p*-Value
Serum IgA (g/L)	1.57 ± 0.29	1.39 ± 0.29	0.242
Serum IgG (g/L)	34.10 ± 14.34	27.33 ± 6.17	0.113
Serum IgM (g/L)	3.30 ± 0.64 ^b^	4.03 ± 0.87 ^a^	0.013
Ileum IgA (g/gprot)	0.77 ± 0.16	0.74 ± 0.13	0.640
Ileum SIgA (mg/gprot)	7.83 ± 3.27	9.14 ± 2.50	0.213

IgA = immunoglobulin A; IgG = immunoglobulin G; IgM = immunoglobulin M; SIgA = secretory immunoglobulin A. ^1^ Data represent the mean value of 8 hens each treatment (*n* = 8). ^a,b^ Means within a row with no common superscripts differ significantly (*p* < 0.05).

**Table 9:** Effects of Compound Probiotic Preparation on Cecal Short-Chain Fatty Acids (SCFAs) Contents of Broiler Breeders During Post-Molting Period ^1^.

Items	Control Group	Probiotic Group	*p*-Value
Acetic Acid (μg/g)	1539.03 ± 127.55	1657.34 ± 119.74	0.088
propionic Acid (μg/g)	741.41 ± 92.46	832.71 ± 109.92	0.118
Butyric Acid (μg/g)	264.70 ± 46.51 ^b^	308.71 ± 27.93 ^a^	0.048
Isobutyric Acid (μg/g)	145.47 ± 41.30 ^b^	187.72 ± 31.37 ^a^	0.037
2-Methylbutyric Acid (μg/g)	94.83 ± 25.59	115.86 ± 33.23	0.178
Valeric Acid (μg/g)	88.72 ± 47.68	93.04 ± 35.71	0.848
Isovaleric Acid (μg/g)	141.74 ± 31.54	171.16 ± 52.66	0.197
4-Methylvaleric Acid (μg/g)	6.15 ± 1.37	4.59 ± 1.85	0.083
Hexanoic Acid (μg/g)	1.79 ± 0.66	1.86 ± 0.64	0.826

^1^ Data represent the mean value of 8 hens each treatment (*n* = 8). ^a,b^ Means within a row with no common superscripts differ significantly (*p* < 0.05).

## Data Availability

The datasets presented in this article are not readily available because the data are part of an ongoing study. Requests to access the datasets should be directed to author Bowen Yang (E-mail: yangbowen@hebau.edu.cn).
